# Higher Serum Immunoglobulin G3 Levels May Predict the Development of Multiple Sclerosis in Individuals With Clinically Isolated Syndrome

**DOI:** 10.3389/fimmu.2018.01590

**Published:** 2018-07-13

**Authors:** Stephanie Trend, Anderson P. Jones, Lilian Cha, Scott N. Byrne, Sian Geldenhuys, Marzena J. Fabis-Pedrini, William M. Carroll, Judith M. Cole, David R. Booth, Robyn M. Lucas, Allan G. Kermode, Martyn A. French, Prue H. Hart

**Affiliations:** ^1^Telethon Kids Institute, University of Western Australia, Perth, WA, Australia; ^2^Sydney Medical School, Westmead Institute for Medical Research, University of Sydney, Westmead, NSW, Australia; ^3^Centre for Neuromuscular and Neurological Disorders, Perron Institute for Neurological and Translational Science, Sir Charles Gairdner Hospital, University of Western Australia, Perth, WA, Australia; ^4^St John of God Dermatology Clinic, St John of God Hospital, Perth, WA, Australia; ^5^National Centre for Epidemiology & Population Health, Research School of Population Health, Australian National University, Canberra, ACT, Australia; ^6^Centre for Ophthalmology and Visual Science, University of Western Australia, Perth, WA, Australia; ^7^Institute for Immunology and Infectious Disease, Murdoch University, Perth, WA, Australia; ^8^UWA Medical School and School of Biomedical Sciences, University of Western Australia, Perth, WA, Australia

**Keywords:** clinically isolated syndrome, multiple sclerosis, immunoglobulin, antibody, biomarkers

## Abstract

Clinically isolated syndrome (CIS) is a first episode of neurological symptoms that may precede a diagnosis of multiple sclerosis (MS). Therefore, studying individuals with CIS may lead to breakthroughs in understanding the development and pathogenesis of MS. In this study, serum levels of immunoglobulin (Ig)G, IgA, IgM, and IgG1–4 were measured in 20 people with CIS and compared with those in 10 healthy controls (HC) and 8 people with MS. Serum Ig levels in individuals with CIS were compared with (a) the time to their conversion from CIS to MS, (b) serum levels of antibodies to Epstein–Barr virus, (c) frequencies of T regulatory (Treg), T follicular regulatory (Tfr), and B cell subsets, and (d) Treg/Tfr expression of Helios. Serum IgG, IgM, and IgG2 levels were significantly lower in people with CIS than HC, and IgG, IgM, and IgG1 levels were significantly lower in people with CIS than MS. After adjusting for age, sex, and serum 25(OH) vitamin D_3_ [25(OH)D] levels, CIS was associated with lower serum levels of IgG and IgG2 compared with HC (*p* = 0.001 and *p* < 0.001, respectively). People with MS had lower IgG2 levels (*p* < 0.001) and IgG2 proportions (%IgG; *p* = 0.007) compared with HC. After adjusting for age, sex, and 25(OH)D, these outcomes remained, in addition to lower serum IgA levels (*p* = 0.01) and increased IgG3 levels (*p* = 0.053) in people with MS compared with HC. Furthermore, serum from people with MS had increased proportions of IgG1 and IgG3 (*p* = 0.03 and *p* = 0.02, respectively), decreased proportions of IgG2 (*p* = 0.007), and greater ratios of “upstream” to “downstream” IgG subclasses (*p* = 0.001) compared with HC. Serum IgG3 proportions (%IgG) from people with CIS correlated with the frequency of plasmablasts in peripheral blood (*p* = 0.02). Expression of Helios by Treg and Tfr cell subsets from individuals with CIS correlated with levels of serum IgG2 and IgG4. IgG3 levels and proportions of IgG3 (%IgG) in serum at CIS diagnosis were inversely correlated with the time until conversion to MS (*p* = 0.018 and *p* < 0.001, respectively), suggesting they may be useful prognostic markers of individuals with CIS who rapidly convert to MS.

## Introduction

Clinically isolated syndrome (CIS) is often a precursor to multiple sclerosis (MS), an immune-mediated inflammatory and degenerative neurological condition with complex etiology involving both genetic and environmental factors. Almost all people with MS have evidence of past infection with Epstein–Barr virus (EBV) (1). Smoking and low past exposure to ultraviolet radiation are also environmental risk factors for MS (2). The disease course following a diagnosis of CIS varies greatly between individuals, with the majority eventually converting to MS (3).

There are currently no reliable biomarkers available to indicate prognosis at CIS presentation, though earlier age at onset, having more lesions detected by magnetic resonance imaging (MRI) at diagnosis, and presence of oligoclonal bands in the cerebrospinal fluid (CSF) are associated with an increased absolute risk of conversion from CIS to MS (4–6). Earlier treatment is associated with decreased morbidity (7), and, as such, identifying predictive biomarkers for disease course early in MS is a research priority (8). Identification of peripheral blood biomarkers that indicate the expected length of time until conversion to MS would be valuable, given the relatively low invasiveness of blood compared with CSF sampling (9). New biomarkers of MS could also identify mediators of inflammation in early disease, which is important since the exact cause of MS is unknown.

While many cellular and humoral immune mediators may be associated with early inflammatory disease in MS, B cells and their products are of particular interest. Ectopic lymphoid follicles are found in the central nervous system of some people with MS. These structures contain B cells and immunoglobulin (Ig)G antibodies (10–12). Although antibodies are likely to be important in MS development, unlike other idiopathic inflammatory demyelinating disorders such as neuromyelitis optica, where aquaporin-4-specific IgG antibody levels are a highly sensitive measure to classify disease and predict relapse (13), no disease-specific MS-antigen has been identified. As such, MS appears to be mediated to some extent by heterogeneous auto-antibodies targeted against neurological antigens (14).

In this study, levels of total IgG, IgA, IgM, and IgG subclasses were investigated in the serum of individuals recently diagnosed with CIS and compared with healthy controls (HC) and people with definite MS. We hypothesized that increased levels of one or more Ig isotype would be present in serum from people with CIS and MS compared with HC, and that higher serum Ig levels at the time of CIS diagnosis would be associated with a shorter time to conversion to MS.

## Materials and Methods

### Study Participants

This study involved participants of the PhoCIS study (*n* = 20), a trial of narrowband UVB phototherapy (311–312 nm) for the prevention of conversion from CIS to MS in a high-risk cohort (15). CIS participants were <120 days from their first demyelinating event at study enrollment. All participants with CIS were drug–naïve and blood samples were collected prior to the UVB phototherapy intervention. Participants were followed up at 3, 6, and 12 months after enrollment using MRI to detect conversion to MS.

Samples from eight people with definite MS and recent clinical symptoms who had not been treated with disease-modifying therapies or steroids in the previous 30 days were collected as a comparison group. Seven MS individuals were newly diagnosed (venepuncture within 22 days of diagnostic MRI) and one individual was diagnosed with MS 7 years ago with a recent MRI relapse. Both CIS and MS were diagnosed according to the 2010 McDonald diagnostic criteria (16), with CIS meeting or exceeding PatyA or PatyB criteria.

In addition, a cohort of non-CIS control participants (*n* = 10) were recruited to examine seasonal serum vitamin D levels (HC) and utilized as a comparison group. The HC had no history of autoimmune disease.

This study was carried out in accordance with the recommendations of the National Health and Medical Research Council of Australia’s National Statement on Ethical Conduct in Human Research. The PhoCIS study protocol was approved by the Bellberry Human Research Ethics committee (2014-02-083) and endorsed by the Human Research Ethics Office of the University of Western Australia (RA/4/1/6796), and the study of MS participants was approved by Sir Charles Gairdner Hospital Human Research Ethics Committee (2006-073). All participants gave written informed consent in accordance with the Declaration of Helsinki prior to study procedures being performed.

### Blood Samples

Peripheral venous blood was collected from individuals with CIS and extensive phenotypes of peripheral blood mononuclear cells (PBMCs) were obtained by multicolor flow cytometry as previously described (17, 18). Blood was also collected in Vacutainer SST tubes (Becton Dickinson, North Ryde, NSW, Australia) and serum separated by centrifugation at 800 × *g* for 10 min, then stored at −80°C until batch analyses.

Serum was also collected in the same manner from HC monthly for 12 months, and as a one-off sample from individuals with MS.

### Serum Ig Assays

Total IgG, IgG2, IgG3, IgG4, total IgA, and IgM were measured using cytometric bead arrays (BD Biosciences, North Ryde, NSW, Australia), with data captured using the BD LSRFortessa flow cytometer, according to the manufacturer’s instructions. IgG2, IgG3, and IgG4 levels were calculated both as absolute concentrations (μg/mL) and as a percentage of total IgG. Since IgG1 was not measured in the bead array assay (due to it becoming unavailable in the BD product range), IgG1 levels and proportions (as % of total IgG) were calculated by subtracting the measured concentrations of IgG2, IgG3, and IgG4 from total IgG in the cytometric bead arrays.

The reliability of the calculated IgG1 levels was confirmed by measuring IgG1 levels using enzyme-linked immunosorbent assays (ELISA; Abcam, Melbourne, VIC, Australia). Although the IgG1 values determined by each method were significantly correlated (intraclass correlation = 0.542, *p* = 0.02), the levels of IgG1 reported by ELISA were consistently lower than expected. Therefore, IgG1 levels and proportion data (%IgG) presented here were those calculated from results of the cytometric bead array assays.

### Measurement of Serum 25(OH) Vitamin D_3_ and EBV Serology

Serum 25(OH) vitamin D_3_ [25(OH)D] was measured by liquid chromatography tandem mass spectroscopy (19). The concentration of anti-EBV antibodies (anti-Viral Capsid Antigen IgG, Anti-EBV nuclear antigen-1 IgG, and anti-EBV IgM) was measured in serum from people with CIS and MS by PathWest Laboratory Medicine (Nedlands, WA, Australia).

### B Cell, T Regulatory (Treg), and T Follicular Regulatory (Tfr) Cell Subset Frequencies in CIS Individuals

B cell, Treg, and Tfr subset frequencies and Treg/Tfr levels of Helios expression in the blood samples from 18 of the 20 individuals in the CIS cohort were previously reported (17, 18). In this study, data from two additional blood donors with CIS were included and analyzed as previously described.

### Statistical Analyses

The data presented for HC [Ig and 25(OH)D] were mean values calculated from serial blood samples collected monthly for 12 months. Data were examined by season (Summer = December–February, Autumn = March–May, Winter = June–August, and Spring = September–November). No significant differences between seasonal Ig levels were detected in HC serum collected over 1 year using Friedman tests (Figure S1 in Supplementary Material), but seasonal serum 25(OH)D levels were significantly lower in winter months than in summer months (data not shown).

Spearman’s rho (ρ) was used to test the correlation between the Ig levels and other continuous variables. Mann–Whitney *U* tests were used to compare continuous outcomes in people with CIS, MS, and HC, and Fisher’s exact test was used for categorical variables. The ratio of “upstream”:“downstream” IgG subclasses was calculated by adding the frequencies of measured IgG3 (%IgG) and calculated IgG1 (%IgG) together and dividing by the combined frequencies of measured IgG2 (%IgG) and measured IgG4 (%IgG).

Correlation between ELISA and cytometric bead array-determined values of serum IgG1 was tested using intraclass correlation, with the average measures intraclass correlation coefficient reported.

Generalized linear models were used to examine the effects of CIS and MS, compared with HC, on Ig levels, adjusting the data for 25(OH)D levels, sex, and age. Data analyses were performed using SPSS software (IBM Corp., v24). For all analyses, a *p*-value <0.05 was considered to be statistically significant.

## Results

### Demographics of Participating Blood Donors

The demographics of the HC and people with CIS and MS are shown in Table 1. Individuals in each group were similar in sex and age, but people with CIS had significantly higher serum 25(OH)D levels than HC (Table 1). All individuals with CIS and MS had evidence of past EBV infection, indicated by positive IgG serology tests. Eighteen people with CIS had no detectable anti-EBV IgM in serum, and two had equivocal levels, suggesting that there were no cases of recent EBV infection. Of the eight individuals with definite MS, six had no detectable anti-EBV IgM in their serum, one had a positive test, and one had an equivocal result.

**Table 1:** Characteristics of the healthy controls (HC), people with clinically isolated syndrome (CIS) or multiple sclerosis (MS) included in the dataset.

	HC (*n* = 10)	CIS (*n* = 20)	MS (*n* = 8)	*p*-Value
Age [median (minimum–maximum)]	35.4 (25.5–46.9)	38.0 (23.4–54.3)	45.0 (18.4–54.6)	0.64
Female sex [*n* (%)]	8 (80)	12 (60)	5 (62.5)	0.54
Serum 25(OH)D (nmol/L) [median (minimum–maximum)]	67.7 (37.9–84.9)*	88.5 (43.7–135.6)*	76.5 (40.8–108.2)	**0.04**
**EBV status**				
Anti-viral capsid antigen IgG [U/mL; median (minimum–maximum)]	ND	289 (73.8–750^#^)	550.5 (72.8–750^#^)	0.12
Anti-EBV nuclear antigen-1 IgG [U/mL; median (minimum–maximum)]	ND	600 (159–600^#^)	426 (38.8–600^#^)	0.86
Time since diagnosis of CIS or MS (days) [median (minimum–maximum)]	n/a	36.5 (−1 to 94)*	6 (0–2,537)*	**<0.001**

*Superscript letter indicates groups which were significantly different to one another in post-test*.

*Bold font and shading indicates a statistically significant p value*.

*Lower limit of detection of assay.

*^#^Upper limit of detection of assay*.

*n/a, not applicable; ND, not done; EBV, Epstein–Barr virus*.

### Different Serum Ig Profiles Between HC, and CIS and MS Individuals

**Figure 1:**
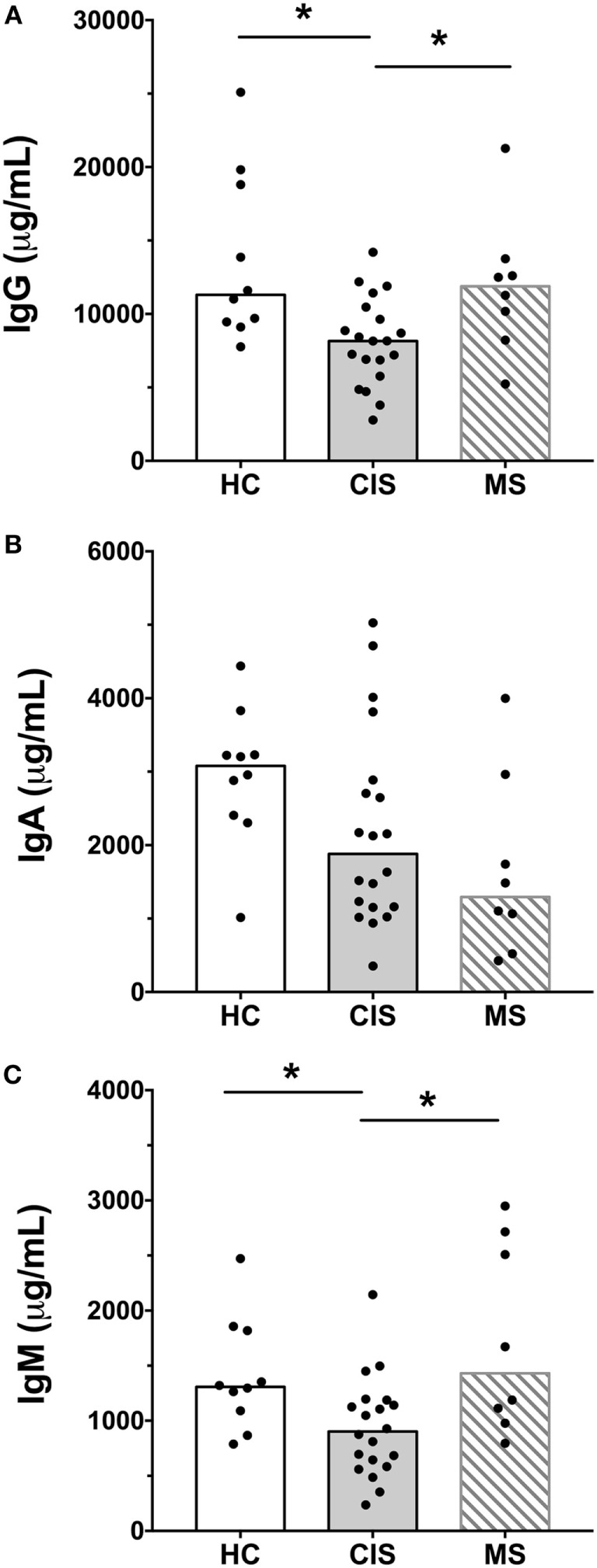
Total IgG **(A)**, IgA **(B)**, and IgM **(C)** concentrations in serum from healthy controls (HC) averaged over a 1-year collection period (*n* = 10; open bars) compared with people with recently diagnosed clinically isolated syndrome (CIS) (*n* = 20; shaded bars) and multiple sclerosis (MS) (*n* = 8, diagonal lines in bars). Individual values are shown and group medians are indicated by the horizontal bar. Statistically significant comparisons in Mann–Whitney tests (*p* < 0.05) are indicated with as asterisk.

**Figure 2:**
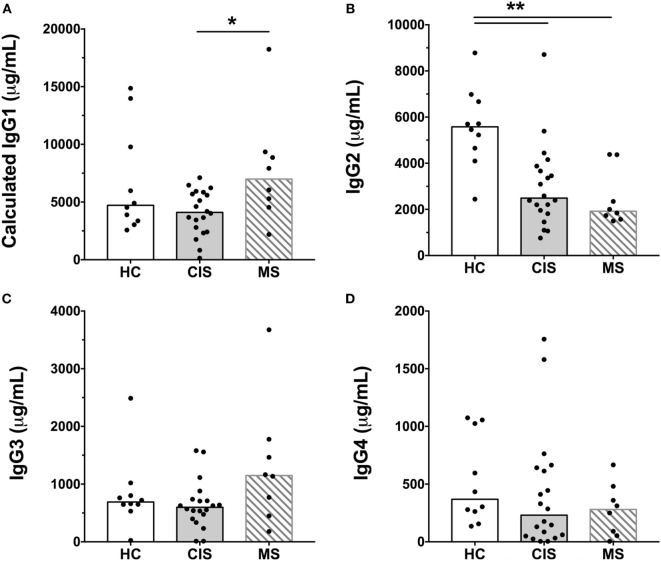
Serum concentrations of IgG subclasses, **(A)** IgG1, **(B)** IgG2, **(C)** IgG3, and **(D)** IgG4 from healthy controls (HC) averaged over a 1-year collection period (*n* = 10; open bars) compared with people with recently diagnosed clinically isolated syndrome (CIS) (*n* = 20; shaded bars) and multiple sclerosis (MS) (*n* = 8, diagonal lines in bars). Individual values are shown and group medians are indicated by the horizontal bar. Statistically significant comparisons in Mann–Whitney tests (*p* < 0.05) are indicated with as asterisk and very significant comparisons (*p* < 0.01) are indicated with two asterisks.

**Figure 3:**
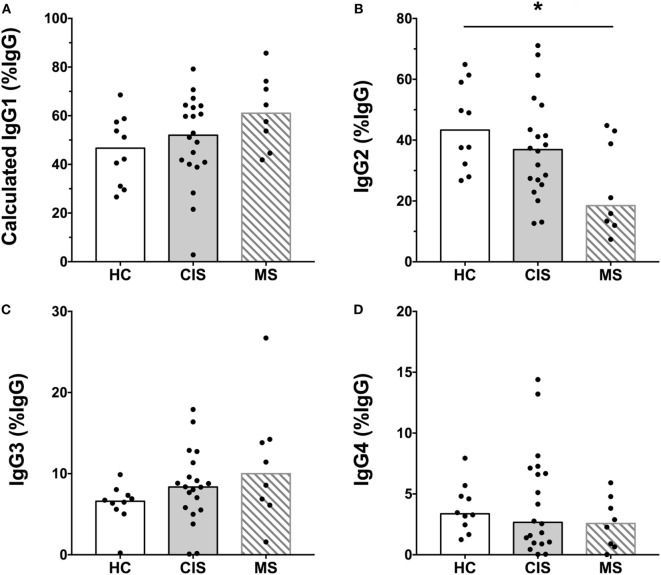
Proportions of calculated **(A)** IgG1, **(B)** IgG2, **(C)** IgG3, and **(D)** IgG4 subclasses as a percent of total IgG shown in Figure 1 from healthy controls (HC) (*n* = 10; open bars) compared with people with recently diagnosed clinically isolated syndrome (CIS) (*n* = 20; shaded bars) and multiple sclerosis (MS) (*n* = 8, diagonal lines in bars). Individual values are shown and group medians are indicated by the horizontal bar. Statistically significant comparisons in Mann–Whitney tests (*p* < 0.05) are indicated with as asterisk.

**Figure 4:**
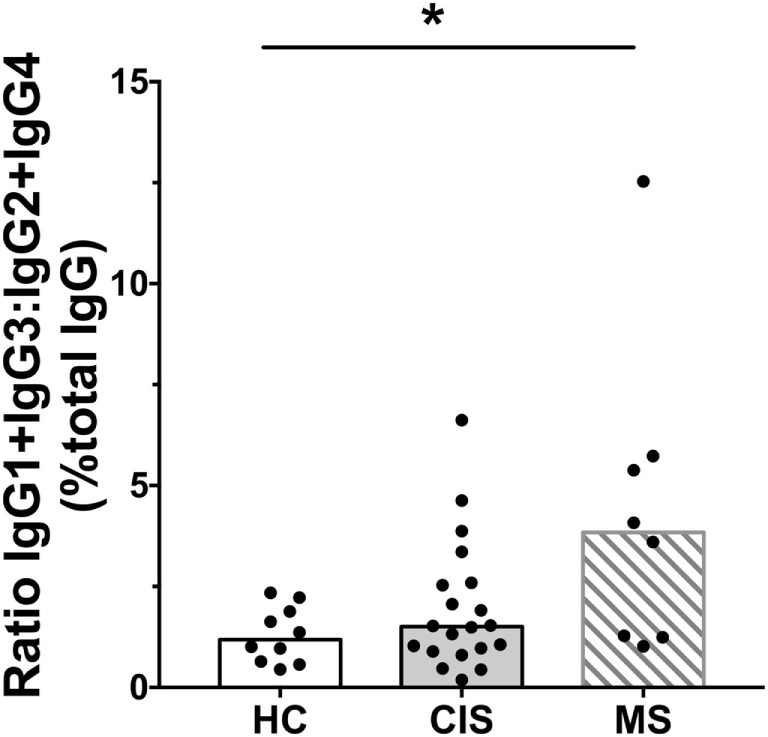
Ratios of calculated IgG1 (%IgG) + IgG3 (%IgG) to IgG2 (%IgG) + IgG4 (%IgG) in serum from healthy controls (HC) averaged over a 1-year collection period (*n* = 10; open bars) compared with people with recently diagnosed clinically isolated syndrome (CIS) (*n* = 20; shaded bars) and multiple sclerosis (MS) (*n* = 8, diagonal lines in bars). Individual values are shown and group medians are indicated by the horizontal bar. Statistically significant comparisons in Mann–Whitney tests (*p* < 0.05) are indicated with as asterisk.

The levels of total measured Ig isotypes, IgG subclasses, and IgG subclasses as proportions of total IgG are shown in Figures 1A–C, Figures 2A–D, and Figures 3A–D, respectively. There were significantly lower serum levels of total IgG and IgM in people with CIS relative to both HC and people with MS (Figures 1A,C). In both the CIS and MS groups, serum IgA levels were non-significantly lower than in HC (*p* = 0.07 and *p* = 0.06, respectively; Figure 1B). When subclasses of IgG were examined, levels of IgG1 were significantly higher in people with MS than those with CIS (Figure 2A), and IgG2 levels were significantly lower in serum from people with both CIS and MS compared with HC (Figure 2B). In people with MS, IgG2 proportions (%IgG) were also significantly lower than in HC (Figure 3B). The relative proportions of IgG1, IgG3, and IgG4 and absolute levels of IgG3 and IgG4 (as %IgG) were not significantly different between groups. However, the ratios of “upstream” (IgG3 + IgG1) to “downstream” (IgG2 + IgG4) IgG subclasses were significantly higher in the MS group than in HC (Figure 4).

As the CIS and HC blood donors had different serum 25(OH)D levels (Table 1), and Ig levels can differ according to age and sex (20), the differences in Ig levels detected between the groups could have been confounded by these variables. Therefore, generalized linear models were used to compare Ig levels between the CIS and MS individuals and HC while simultaneously adjusting for these other potentially related predictor variables. When Ig data were adjusted for serum 25(OH)D levels, sex and age in the models, total IgG in CIS and IgG2 levels in both CIS and MS remained significantly lower than those in HC (Table 2), confirming the results of the univariable analyses. Differences in serum IgM levels between CIS and HC detected using the raw data were not significant after adjusting data for age, sex, and 25(OH)D. Total IgA levels remained non-significantly lower in CIS compared with HC (*p* = 0.069) but unlike in the univariate comparison, relative to HC, people with MS were associated with significantly lower serum IgA levels and significantly higher IgM levels after adjusting for other variables. IgG3 levels in people with MS were non-significantly increased compared with HC (*p* = 0.053). Furthermore, after adjusting for age, sex, and 25(OH)D, MS was associated with significantly increased proportions of IgG1 and IgG3, and decreased proportions of IgG2 (as % of total IgG), resulting in a significantly greater ratio of “upstream” to “downstream” IgG subclasses.

**Table 2:** Results of regression analyses for serum immunoglobulin levels, adjusting for clinical variables.

Outcome variable	Independent variable	Unadjusted estimate	95% Confidence interval		*p*-Value
			Lower	Upper	
Total IgG (μg/mL)	MS vs. HC	−1,703.39	−5,511.1	2,104.33	0.38
	CIS vs. HC	−5,574.52	−8,966.63	−2,182.41	**0.001**
	Male vs. female sex	−606.79	−3,324.34	2,110.76	0.66
	Age	−57.68	−202.69	87.33	0.44
	25(OH)D	13.99	−45.57	73.56	0.65

IgA (μg/mL)	MS vs. HC	−1,352.97	−2,427.85	−278.09	**0.014**
	CIS vs. HC	−887.93	−1,845.49	69.63	0.069
	Male vs. female sex	259.36	−507.78	1,026.50	0.51
	Age	−31.69	−72.63	9.24	0.13
	25(OH)D	6.652	−10.16	23.47	0.44

IgM (μg/mL)	MS vs. HC	502.84	54.34	951.34	**0.028**
	CIS vs. HC	−209.65	−609.19	189.90	0.30
	Male vs. female sex	−494.65	−814.74	−147.55	**0.002**
	Age	−2.38	−19.46	14.70	0.785
	25(OH)D	−6.03	−13.05	0.99	0.09

Calculated IgG1 (μg/mL)	MS vs. HC	1,213.67	−1,957.62	4,384.96	0.45
	CIS vs. HC	−2,523.73	−5,348.88	301.42	0.08
	Male vs. female sex	−591.83	−2,855.17	1,671.51	0.61
	Age	−25.95	−146.72	94.82	0.67
	25(OH)D	4.75	−44.86	54.36	0.85

IgG2 (μg/mL)	MS vs. HC	−3,177.1	−4,718.33	−1,635.86	**<0.001**
	CIS vs. HC	−2,693.94	−4,066.95	−1,320.93	**<0.001**
	Male vs. female sex	161.04	−938.94	1,261.01	0.77
	Age	−7.50	−66.19	51.20	0.8
	25(OH)D	4.48	−19.63	28.59	0.72

IgG3 (μg/mL)	MS vs. HC	544.22	−7.10	1,095.54	0.053
	CIS vs. HC	−162.53	−653.67	328.62	0.52
	Male vs. female sex	−286.54	−680.01	106.94	0.15
	Age	−24.77	−45.76	−3.77	**0.02**
	25(OH)D	4.14	−4.48	12.77	0.35

IgG4 (μg/mL)	MS vs. HC	−284.18	−635.05	66.69	0.11
	CIS vs. HC	−194.32	−506.89	118.25	0.22
	Male vs. female sex	110.54	−139.88	360.95	0.39
	Age	0.53	−12.83	13.89	0.94
	25(OH)D	0.62	−4.87	6.11	0.83

Calculated IgG1 (%IgG)	MS vs. HC	16.25	0.84	31.66	**0.03**
	CIS vs. HC	3.74	−9.99	17.47	0.59
	Male vs. female sex	−2.49	−13.49	8.51	0.66
	Age	−0.08	−0.66	0.51	0.80
	25(OH)D	0.06	−0.19	0.30	0.66

IgG2 (%IgG)	MS vs. HC	−19.9	−34.33	−5.47	**0.007**
	CIS vs. HC	−6.29	−19.14	6.56	0.34
	Male vs. female sex	1.20	−9.10	11.5	0.82
	Age	0.19	−0.36	0.74	0.51
	25(OH)D	−0.06	−0.28	0.17	0.62

IgG3 (%IgG)	MS vs. HC	5.00	0.69	9.31	**0.02**
	CIS vs. HC	2.14	−1.70	5.98	0.28
	Male vs. female sex	−0.33	−3.40	2.75	0.84
	Age	−0.16	−0.33	0.003	0.054
	25(OH)D	0.02	−0.05	0.09	0.60

IgG4 (%IgG)	MS vs. HC	−1.36	−4.36	1.64	0.38
	CIS vs. HC	0.41	−2.26	3.08	0.76
	Male vs. female sex	1.62	−0.52	3.76	0.14
	Age	0.05	−0.06	0.16	0.39
	25(OH)D	−0.02	−0.06	0.03	0.52

Ratio of IgG1 + IgG3:IgG2 + IgG4	MS vs. HC	3.09	1.18	4.99	**0.001**
	CIS vs. HC	0.60	−1.10	2.30	0.49
	Male vs. female sex	−0.45	−1.81	0.91	0.52
	Age	−0.01	−0.09	0.06	0.74
	25(OH)D	0.01	−0.02	0.04	0.54

*Table shows unadjusted parameter estimates with 95% CIs*.

*Statistically significant p-values are indicated by bolded font and shading*.

*p-Values <0.05 indicates a p-value between 0.01 and 0.05*.

*CI, confidence interval; CIS, clinically isolated syndrome; HC, healthy controls; MS, multiple sclerosis*.

Combining all participant groups and adjusting for the effects of CIS, sex, and serum 25(OH)D levels, absolute IgG3 levels were significantly inversely associated with age, and lower IgM levels with male sex. There were no associations between Ig levels and 25(OH)D levels.

In summary, compared with HC after adjustment for possible confounders, CIS was associated with lower serum levels of total IgG and IgG2. MS was associated with lower serum levels of IgG2 and IgA, increased levels of IgM, decreased proportions of IgG2, and increased proportions of “upstream” IgG subclasses IgG3 and IgG1.

### Serum IgG3 Levels Inversely Correlated With Time to MS Conversion

**Figure 5:**
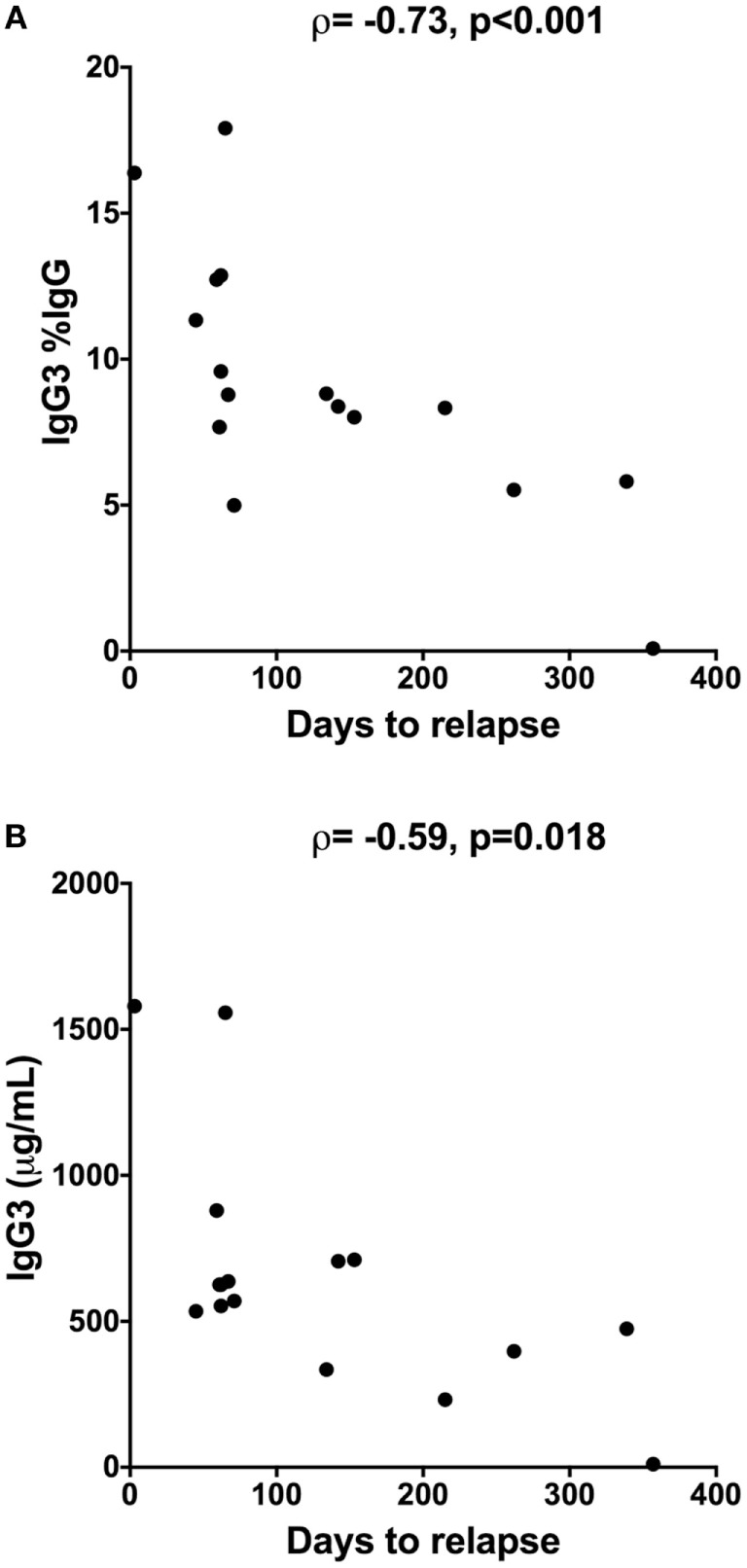
Inverse correlation between time to relapse [conversion from clinically isolated syndrome (CIS) to multiple sclerosis] and **(A)** proportions of IgG3 (%IgG) or **(B)** IgG3 levels in serum from people with CIS (*n* = 16). Serum was collected within 120 days of CIS diagnosis and individuals were followed for 12 months using magnetic resonance imaging to determine date of relapse. IgG3 was measured using cytometric bead arrays. Spearman correlation is shown using Spearman’s rho (ρ), alongside *p*-values.

Since prognostic markers of conversion to MS would be highly valuable in people with newly diagnosed CIS, correlations were investigated between the serum Ig levels of people with CIS and the number of days until conversion to MS. Conversion from CIS to MS within 12 months of collection of blood was confirmed by MRI in 16 of the 20 people with CIS. 3 of the 20 people with CIS had not converted after 12 months, and one was lost to follow-up after 2 months (15). Analyses of serum samples from the individuals with known outcomes showed that higher levels of IgG3 (Figure 5A) and a higher proportion of IgG3 (as %IgG; Figure 5B) in serum close to CIS diagnosis were inversely correlated with the number of days to conversion from CIS to MS (Figure 5). This association was maintained for both predictors when adjusted for serum 25(OH)D levels, age, and sex in regression analyses (data not shown). A weaker positive correlation was also observed for serum IgG2 levels and time to conversion (ρ = 0.52, *p* = 0.04), but the association was not maintained in regression analyses adjusting for 25(OH)D levels, age, and sex.

### Correlations Between Serum EBV-Specific Antibodies and Serum Ig Levels

Since elevated IgG antibody levels against EBV are associated with an increased MS risk (21) and EBV-specific antibodies are predominantly of the IgG1 and IgG3 subclasses (22), serum levels of antibodies against EBV were investigated for correlations with Ig levels, including IgG3. No correlations were found between with the levels of total IgG, IgA, IgM, or IgG subclasses and EBV antibodies in serum from people with CIS (data not shown).

### Correlations Between Serum Ig and PBMC Subset Frequencies and Helios Expression in Tregs

Earlier work from this laboratory investigated differences in PBMC subsets between HC (a different cohort than those reported here) and those with CIS (17, 18). The frequencies of transitional B cells and CD1c + B cells were increased in people with CIS compared with HC (18). Treg and Tfr cells were categorized into different fractions according to FoxP3 and CD45RA expression (17), which is a more advanced dissection of Tregs originally described by Sakaguchi and colleagues (23), as well as by their expression of Helios, a key transcription factor associated with Treg functions (24). Treg and Tfr cell frequencies and Treg/Tfr cells with low Helios expression were identified as subsets differentiating CIS from HC. Therefore, B cell subsets and Treg/Tfr subsets were tested for correlation with levels of serum Ig in people with CIS.

#### B Cell Frequencies

Serum Ig concentrations in people with CIS were tested for correlation with the frequencies of total B cells, transitional B cells, memory B cells, and plasmablasts in peripheral blood (cellular phenotypes are described in Table 3). There was no correlation between the frequency of total B cells (%PBMC) and the concentrations of any Ig in serum (data not shown). However, switched memory B cell frequencies (%B cells) positively correlated with IgG1 proportions (%IgG), and inversely correlated with IgG2 proportions (%IgG) (Table 3). In addition, the frequency of plasmablasts (%B cells) was positively correlated with IgG3 proportions (%IgG). There was a non-significant correlation between the proportion of IgG3 (%IgG) and the frequency of double-negative memory B cells (Table 3).

**Table 3:** A comparison of absolute serum levels (μg/mL) and proportions of IgG subclasses (%IgG) vs. %B cells in individuals with clinically isolated syndrome (*n* = 20).

% B cells	Phenotype	Statistic	IgA	IgM	Total IgG	Calculated IgG1	IgG2	IgG3	IgG4	Calculated IgG1% IgG	IgG2% IgG	IgG3%IgG	IgG4% IgG
Transitional B cells	CD19^+^CD20^+^CD27^−^IgD^+^CD24^hi^CD38^hi^	ρ	−0.25	−0.28	−0.41	−0.40	−0.12	−0.36	0.15	−0.10	0.14	0.04	0.28
		*p*-Value	0.30	0.24	0.08	0.08	0.63	0.12	0.53	0.65	0.56	0.86	0.23

Switched memory B cells	CD19^+^CD20^+^CD27^+^IgD^−^	ρ	−0.25	−0.23	0.10	0.23	−0.34	0.08	−0.17	**0.55**	**−0.47**	−0.01	−0.24
		*p*-Value	0.28	0.33	0.68	0.33	0.14	0.73	0.47	**0.01**	**0.04**	0.97	0.31

Non-switched memory B cells	CD19^+^CD20^+^CD27^+^IgD^+^	ρ	−0.23	−0.28	0.06	0.04	−0.03	0.23	−0.01	−0.04	−0.07	0.05	0.03
		*p*-Value	0.33	0.23	0.82	0.86	0.89	0.32	0.96	0.88	0.76	0.85	0.91

CD27 + memory B cells	CD19^+^CD20^+^CD27^+^	ρ	−0.31	−0.41	0.10	0.10	−0.17	0.27	−0.05	0.15	−0.26	0.06	−0.05
		*p*-Value	0.19	0.08	0.68	0.68	0.48	0.25	0.84	0.51	0.27	0.79	0.84

Plasmablasts	CD19^+^CD20^−^CD38^hi^CD27^hi^IgD^−^CD24^−^	ρ	0.34	0.20	−0.04	0.03	−0.35	0.37	0.10	0.13	−0.28	**0.52**	0.12
		*p*-Value	0.14	0.15	0.88	0.91	0.13	0.11	0.67	0.59	0.24	**0.02**	0.62

Double negative memory B cells	CD19^+^CD20^+^IgD^−^CD27^−^	ρ	−0.25	−0.07	−0.15	−0.11	−0.35	0.19	−0.12	0.35	−0.28	0.44	−0.04
		*p*-Value	0.30	0.77	0.53	0.64	0.13	0.43	0.63	0.14	0.24	0.06	0.88

*Statistically significant correlation is shown in bolded values with shading*.

*p-Values <0.05 indicates a p-value between 0.01 and 0.05*.

#### Treg and Tfr Cell Frequencies and Helios Expression

Total IgG levels were inversely correlated with cytokine-producing non-Treg (FrIII) frequencies, as shown in Table 4 (cellular phenotypes are described in Table 4). The expression of Helios (quantified as geometric mean fluorescence intensity; MFI) in several Treg and Tfr fractions was significantly correlated with total IgG, IgG2, and IgG4 in serum (Table 5). However, IgG3 subclass levels were independent of Helios expression in people with CIS.

**Table 4:** A comparison of absolute serum levels (μg/mL) and proportions of IgG subclasses (%IgG) vs. %Treg or %Tfr cells in individuals with clinically isolated syndrome (*n* = 20).

	Phenotype	Statistic	IgA	IgM	Total IgG	Calculated IgG1	IgG2	IgG3	IgG4	Calculated IgG1% IgG	IgG2% IgG	IgG3%IgG	IgG4% IgG
FrI Treg (%Treg)	CD3^+^CD4^+^CXCR5^−^FoxP3^lo^CD45RA^+^ Treg	ρ	−0.08	0.39	0.30	0.09	0.13	0.09	0.11	−0.01	−0.03	−0.11	0.06
		*p*-Value	0.73	0.09	0.20	0.74	0.60	0.72	0.65	0.97	0.90	0.64	0.81

FrII Treg (%Treg)	CD3^+^CD4^+^CXCR5^−^FoxP3^hi^CD45RA^−^ Treg	ρ	0.22	−0.13	0.21	0.38	0.08	0.12	0.04	0.16	−0.21	−0.02	0.003
		*p*-Value	0.36	0.60	0.37	0.13	0.74	0.61	0.88	0.50	0.37	0.94	0.99

FrIII non-Treg (%Treg)	CD3^+^CD4^+^CXCR5^−^FoxP3^lo^CD45RA^−^ Treg	ρ	−0.06	−0.20	**−0.57**	−0.30	−0.14	−0.18	−0.01	−0.31	0.39	0.23	0.11
		*p*-Value	0.80	0.39	**<0.01**	0.24	0.57	0.45	0.96	0.19	0.09	0.32	0.65

FrI Tfr (%Tfr)	CD3^+^CD4^+^CXCR5^+^FoxP3^lo^CD45RA^+^Tfr	ρ	−0.07	0.44	0.14	−0.03	0.13	0.12	−0.003	−0.13	0.06	0.07	0.04
		*p*-Value	0.77	0.05	0.57	0.93	0.59	0.60	0.99	0.59	0.82	0.78	0.88

FrII Tfr (%Tfr)	CD3^+^CD4^+^CXCR5^+^FoxP3^hi^CD45RA^−^ Tfr	ρ	0.001	−0.22	0.21	0.12	0.04	0.33	−0.06	0.10	−0.1	0.105	−0.14
		*p*-Value	0.10	0.36	0.37	0.66	0.88	0.15	0.80	0.68	0.52	0.66	0.56

FrIII non-Tfr (%Tfr)	CD3^+^CD4^+^CXCR5^+^FoxP3^lo^CD45RA^−^ Tfr	ρ	0.02	−0.24	−0.36	0.01	−0.21	−0.38	0.13	0.03	0.07	−0.05	0.16
		*p*-Value	0.92	0.31	0.12	0.98	0.37	0.10	0.57	0.89	0.77	0.82	0.51

*Statistically significant correlation is shown in bolded values with shading*.

**Table 5:** A comparison of absolute serum levels (μg/mL) and proportions of IgG subclasses (%IgG) vs. Helios expression on Treg and Tfr cell (median fluorescence intensity; MFI) in individuals with clinically isolated syndrome.

	Statistic	IgA	IgM	Total IgG	Calculated IgG1	IgG2	IgG3	IgG4	Calculated IgG1% IgG	IgG2% IgG	IgG3% IgG	IgG4% IgG
FrI Treg Helios expression (MFI)	ρ	0.27	−0.21	0.35	0.35	**0.54**	0.15	**0.45**	−0.23	0.24	−0.10	0.38
	*p*-Value	0.26	0.38	0.14	0.17	**0.02**	0.53	**<0.05**	0.34	0.32	0.67	0.10

FrII Treg Helios expression (MFI)	ρ	0.35	−0.21	0.16	0.26	**0.60**	0.05	**0.46**	**−0.50**	**0.49**	−0.007	**0.46**
	*p*-Value	0.12	0.37	0.50	0.32	**0.01**	0.83	**0.04**	**0.03**	**0.03**	0.98	**0.04**

FrIII non-Treg Helios expression (MFI)	ρ	0.37	−0.25	0.21	−0.01	**0.64**	0.03	**0.46**	**−0.48**	**0.49**	−0.08	**0.45**
	*p*-Value	0.11	0.30	0.31	0.99	**<0.01**	0.89	**0.04**	**0.03**	**0.03**	0.74	**<0.05**

FrI Tfr Helios expression (MFI)	ρ	0.34	−0.20	**0.48**	0.39	**0.54**	0.19	**0.49**	−0.15	0.15	−0.17	0.36
	*p*-Value	0.14	0.39	**0.03**	0.12	**0.01**	0.43	**0.03**	0.53	0.53	0.49	0.12

FrII Tfr Helios expression (MFI)	ρ	0.26	−0.16	0.27	0.44	**0.5**	0.22	0.38	−0.29	0.28	0.07	0.32
	*p*-Value	0.27	0.49	0.25	0.08	**0.02**	0.35	0.10	0.21	0.23	0.77	0.17

FrIII non-Tfr Helios expression (MFI)	ρ	0.25	−0.23	0.42	0.19	**0.56**	0.18	0.42	−0.19	0.22	−0.13	0.32
	*p*-Value	0.29	0.33	0.07	0.46	**0.01**	0.45	0.06	0.42	0.36	0.59	0.17

*Statistically significant correlation is shown in bolded values with shading*.

*p-Values <0.05 indicates a p-value between 0.01 and 0.05*.

## Discussion

In a key finding of this work, IgG3 levels and proportions of IgG3 (%IgG) in serum were identified as a potential prognostic marker for people with CIS who rapidly convert to MS. The detection of ≥9 lesions by MRI is a marker of people with CIS who are likely to convert to MS (4). However, in this study, an inverse correlation between serum IgG3 levels and time until MS conversion was observed, suggesting the potential of serum IgG3 to complement the categorical risk assessment from using MRI data, and incorporate the dimension of time in the prognostic assessment when an individual presents with CIS. Moreover, although CSF oligoclonal bands are used as a prognostic marker in MS, most of our CIS participants did not have CSF collected, highlighting the benefit of a developing a blood-based prognostic test for people with CIS. The potential prognostic capacity of serum IgG3 levels in people with CIS should be confirmed in a larger cohort. The strength of this research was the inclusion of individuals with high-risk CIS, where most converted to MS within 12 months of diagnosis. This is a population that is rarely studied but is important to include in research to understand how MS develops.

People with definite MS exhibited higher serum proportions of IgG3 (%IgG) and lower serum IgG2 levels than HC in the adjusted analysis, suggesting that these abnormalities are markers of B cell dysfunction associated with the immunopathogenesis of MS. These findings indicate that although total IgG levels are lower in CIS compared with HC, presumably reflecting IgG2 deficiency, the relative proportions of cells secreting IgG3 may be increased compared with those secreting IgG2 in MS. Serum IgG3 levels in people with CIS correlated with proportions of circulating plasmablasts (CD19^+^CD20^−^CD38^hi^CD27^hi^IgD^−^CD24^−^), cells which are increased in the CSF of people with MS (25). Non-significant correlation between the frequency of CD19^+^CD27^−^IgD^−^ cells (%B cells) and proportion of serum IgG3 (%IgG) was observed in people with CIS. Supporting this finding, cells with a similar CD19^+^CD27^−^IgG^+^ phenotype were reported to produce relatively high amounts of IgG3 and low amounts of IgG2 (26). However, the secretory activities of specific circulating cell subsets were not tested in this study. IgG3 has stronger affinity for the activatory Fcγ receptors, FcγRIIA and FcγRIIIA, on immune cells than the “downstream” subclasses, IgG2 and IgG4 (27, 28). Therefore, IgG3 could actively contribute to systemic pro-inflammatory immune responses in CIS and MS. This study, as well as those demonstrating the different effects of FcγR allele variants on therapeutic responses and prognosis in MS and other immune-mediated diseases (29–32), suggest that the effects of increased IgG3 proportions in serum on the immune cells that express FcγRs warrant further investigation in MS.

Several explanations for the association of higher serum IgG3 proportions with a faster rate of conversion from CIS to MS are worthy of consideration. First, this might reflect a relative predominance of IgG3 antibodies to myelin oligodendrocyte glycoprotein (11) as part of the postulated involvement of auto-antibodies in mediating MS demyelination (33). Second, it might indicate an active antibody response against a viral pathogen, such as EBV, that triggers an immune response against myelin. However, a relationship between serum IgG3 and serum levels of antibodies to EBV was not found in this study. Finally, it is possible that the decreased serum IgG2 levels in people with both CIS and MS, increased proportions of IgG3 and IgG1 in people with MS [after adjustment for age, sex, and 25(OH)D], and the inverse correlation of serum IgG3 proportions (%IgG) with time to conversion from CIS to MS may reflect an acquired immunoregulatory defect, similar to that which has been described in chronic human immunodeficiency virus infection (34).

In people with MS, there was a shift in proportions of IgG subclasses as a percentage of total IgG compared with HC, with increased proportions of “upstream” IgG3 and IgG1 and decreased proportions of the “downstream” IgG2 subclass. No differences between IgG4 levels were detected between groups, perhaps reflecting the minor contribution of this IgG subclass to total IgG levels in serum. Sequential class switch recombination of heavy chain genes during B cell differentiation in germinal center reactions initially results in production of antibodies of “upstream” isotypes IgM, IgG3, and IgG1, followed by those of “downstream” isotypes IgA1, IgG2, and IgG4 (35). Therefore, the combination of increased IgM in MS, increased proportions of IgG3 and IgG1 in MS, and decreased IgG2 and IgA levels in CIS and MS suggest that dysregulation of antibody isotype diversification is occurring in people with high-risk CIS and MS. This may result from decreased B cell maturation activity in germinal centers (36), and subsequent decreases in the frequencies of mature cells producing downstream IgG subclasses. There was an increased frequency of transitional B cells detected in people with CIS compared with HC (18); transitional B cells in this study had some identifying characteristics congruent with the functionally immature B cell phenotype reported to have limited capacity for Ig production (37). Given the importance of CD40 for class switching to occur in germinal centers (38), and the finding of decreased B cell CD40 expression in association with the MS risk allele rs4810485*T (39), we suggest that there may be impairment of normal class switching in CIS and MS mediated, in part, by a reduction in CD40–CD40L engagement on maturing B cells.

It was hypothesized that people with CIS would have increased serum levels of Ig compared with healthy individuals. However, both total IgG and IgM were lower in CIS compared with HC. Although most of the people with CIS converted to MS, low IgM and IgG were not detected in the MS group, suggesting the secretory functions of antibody-secreting cells in people with CIS may be transiently impaired for reasons that are unclear. Reports in the literature on Ig levels in MS have been variable; people with MS have been described to have similar serum Ig levels compared with controls (40), higher IgA and lower IgM in MS compared with HC (41), or lower levels of total IgG and IgG4 (42). The people with CIS and MS in our cohort represent a different population to those in previous studies, where participants had established MS. Moreover, the small number of individuals studied in the MS group may limit the generalizability of these findings to a broader MS cohort, particularly those with long-established disease, who were not well represented in our study.

Given that Tregs can suppress antibody responses (43) and our previous findings indicated that they are functionally impaired in CIS (17), it was proposed that the levels of Treg and Tfr subsets and their relative Helios expression may inversely correlate with Ig levels in serum. Rather, IgG2 and IgG4 levels in serum were positively associated with Helios expression in Treg and Tfr, but IgG3 levels were not related to the extent of Helios expression or to the frequencies of Treg subsets. Future studies should confirm whether the relative increases of IgG3 antibodies in people with high-risk CIS are dependent on changes in Treg functionality or other aspects of immune regulation. Although low serum 25(OH)D has been associated with an increased risk of MS (44), this cohort was characterized by vitamin D sufficiency [25(OH)D > 50 nmol/L] in all but one individual with CIS, and yet most converted to MS. In addition, no associations were found between 25(OH)D and Ig levels in serum, although this study was not designed to specifically investigate this association. IgG production *in vitro* by plasma cells is reported to be limited by 1,25(OH)_2_D_3_ (45). *In vivo*, positive or negative associations between 25(OH)D levels and/or season and IgG levels in serum are variously reported (46–48) but in people with MS, no association between vitamin D and serum or intrathecal IgG has been reported (49, 50). Research intended to investigate the effects of vitamin D metabolites on Ig production in plasma cells from people with MS may resolve whether there is significant interaction between these factors.

The major findings of this study were that lower serum levels of IgG2, and total IgG (which can be partially explained by lower IgG2 levels) were detected in drug–naïve people recently diagnosed with CIS compared with HC, and that there was an association between higher serum IgG3 levels at CIS diagnosis and earlier conversion to MS. The decrease in serum IgG2 was confirmed in people with MS, but in the absence of changes in total IgG levels, this resulted in higher ratios of “upstream” to “downstream” IgG subclasses compared with HC. Based on these findings, it can be hypothesized that impaired antibody class switching in germinal centers might be occurring in people with MS. This study suggests that serum IgG3 levels and/or proportions of total IgG could be an indicator of disease prognosis and might be used to guide the use of future immune therapies in people with CIS. This could be tested in clinical trials. As serum Ig levels in people with CIS did not correlate with levels of anti-EBV antibodies, there appears to be an expansion of IgG3-secreting cells with unknown antigen specificity or clonality which should be investigated.

## Ethics Statement

This study was carried out in accordance with the recommendations of the National Health and Medical Research Council of Australia’s National Statement on Ethical Conduct in Human Research. The PhoCIS study protocol was approved by the Bellberry Human Research Ethics committee (2014-02-083) and endorsed by the Human Research Ethics Office of the University of Western Australia (RA/4/1/6796), and the study of MS participants was approved by Sir Charles Gairdner Hospital Human Research Ethics Committee (2006-073). All participants gave written informed consent in accordance with the Declaration of Helsinki prior to study procedures being performed.

## Author Contributions

ST wrote the first draft of the paper. All the authors contributed to the drafting and editing the manuscript and approved the final version. PH, AK, SB, RL, DB, WC, and JC conceived the idea to perform the analysis of cells and Ig in blood from people with CIS and their subsequent conversion to MS. ST, SB, AJ, and PH designed the experiments. ST, AJ, SG, and LC performed the experiments. MF-P, JC, and AK contributed participant data. ST and AJ analyzed the data. MF contributed to data analysis and manuscript preparation.

## Conflict of Interest Statement

The authors declare that the research was conducted in the absence of any commercial or financial relationships that could be construed as a potential conflict of interest.
