# Supplementary material for: Higher Serum Immunoglobulin G3 Levels May Predict the Development of Multiple Sclerosis in Individuals With Clinically Isolated Syndrome
Source: Front Immunol. 2018 Jul 13;9:1590. doi: 10.3389/fimmu.2018.01590 (PMC6053531; doi:10.3389/fimmu.2018.01590)
Supplement: Supplementary file 1 [file Image_1.PDF]

Supplementary Material

Higher Serum Immunoglobulin G3 Levels May Predict the Development of Multiple Sclerosis in Individuals With Clinically Isolated Syndrome

Stephanie Trend, Anderson P Jones, Lilian Cha, Scott N Byrne, Sian Geldenhuys, Marzena J Fabis-Pedrini, William M Carroll, Judith M Cole, David R Booth, Robyn M Lucas, Allan G Kermode, Martyn A French, Prue H Hart*

*** Correspondence:** Corresponding Author: prue.hart@telethonkids.org.au

1.1 Supplementary Figures

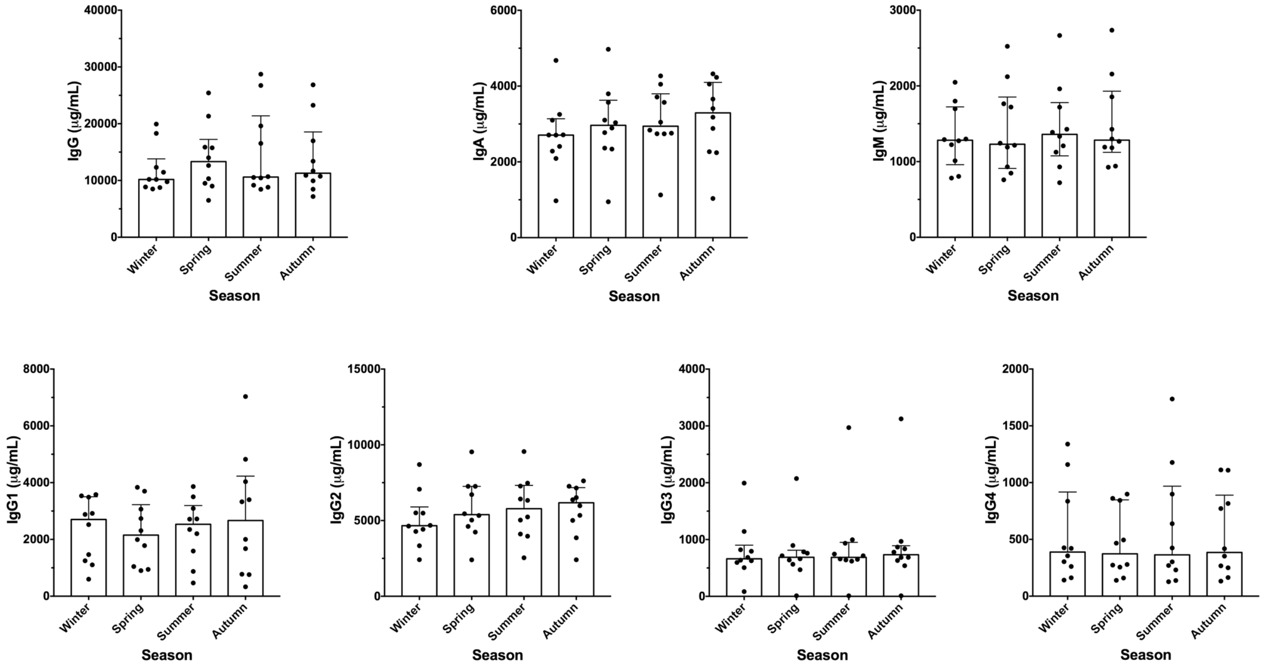

Supplementary Figure 1. Serum immunoglobulin levels across different season in healthy individuals. Monthly blood samples collected from ten healthy individuals were tested for total IgG, total IgA, IgM, and IgG subsets IgG2, IgG3 and IgG4 using a cytometric bead array, and IgG1 was measured using an enzyme-linked immunosorbent assay. Each data point represents the average of three measurements per season per individual (closed circles). Bars and lines indicate the median and interquartile ranges for individual values, respectively. Seasonal effects were compared using Friedman test with Dunn's post−test for multiple comparisons. No significant differences across seasons were detected.
